# Commentary: Cold water swimming reshapes gut microbiome to improve high-fat diet-induced obesity

**DOI:** 10.3389/fmicb.2025.1626745

**Published:** 2025-07-02

**Authors:** Yingjie Liu, Peishan Yu, Keying Wang, Wenjiang Wu

**Affiliations:** Shenzhen Hospital (Futian) of Guangzhou University of Chinese Medicine, Shenzhen, China

**Keywords:** gut microbiome, obesity, cold water swimming, high-fat diet (HFD), commentary

This commentary critiques Men et al.'s (2025) study demonstrating cold water swimming (CWS) alleviates obesity via gut microbiota (GM) remodeling and fecal microbiota transplantation (FMT). While acknowledging the mechanistic insights linking CWS to adipose browning and barrier repair, we highlight critical gaps: unvalidated bacterial functions (e.g., Lachnospiraceae_NK4A136_group), unresolved cold-versus-exercise contributions to AMPK/SIRT1/PGC-1α activation, and overlooked translational hurdles in human applicability. We further propose multi-omics validation and safety frameworks for FMT translation. Addressing these limitations will bridge murine findings to clinically actionable anti-obesity strategies.

## Introduction

The study by Men et al. (2025) investigates the role of cold water swimming (CWS) in ameliorating high-fat diet (HFD)-induced obesity through gut microbiota (GM) remodeling and demonstrates the transferability of these effects via fecal microbiota transplantation (FMT). While the findings highlight CWS as a potential anti-obesity strategy, several methodological and interpretative limitations warrant critical evaluation to advance translational applications.

## Mechanistic and model limitations

**Figure 1:**
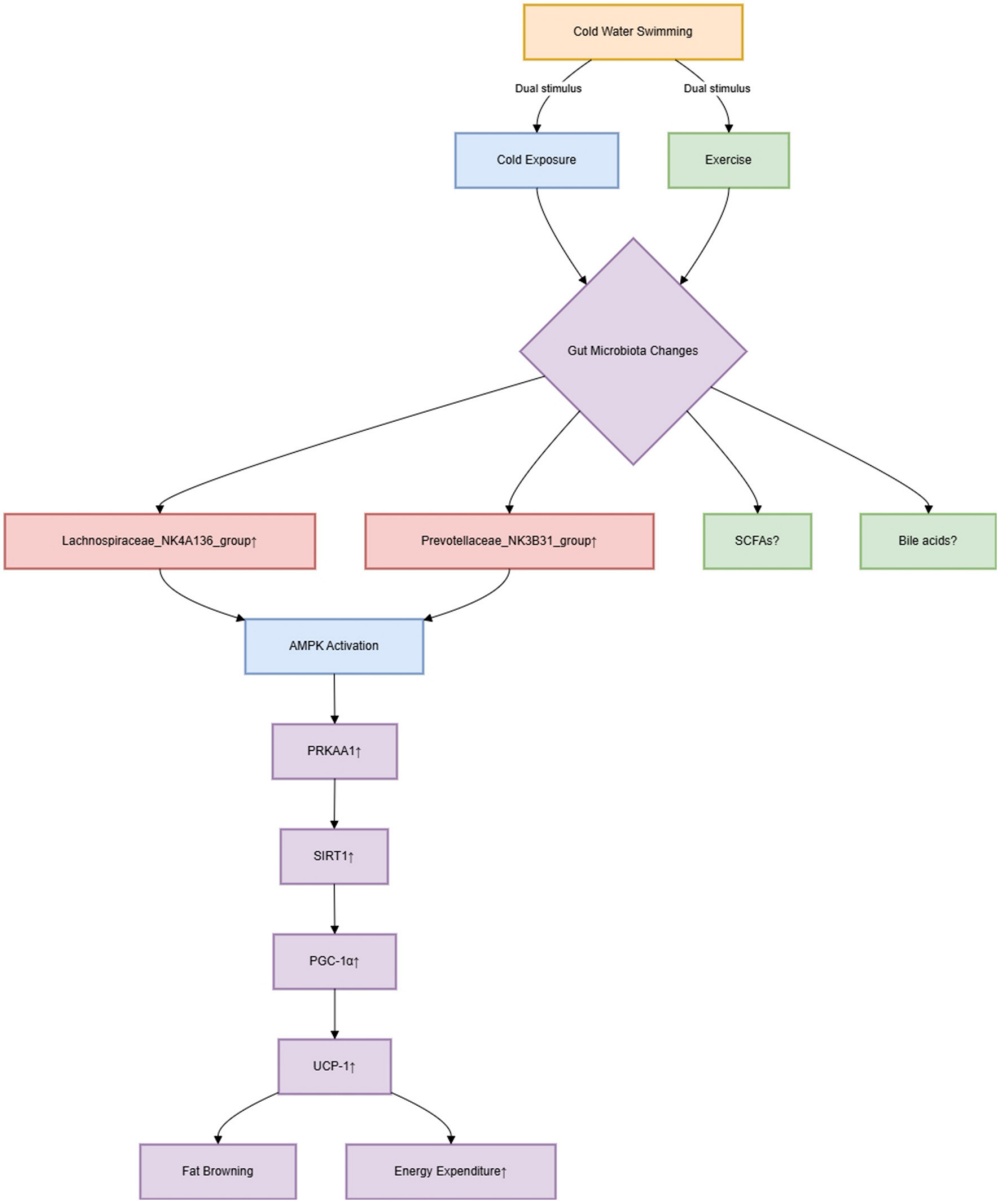
AMPK/SIRT1/PGC-1α pathway and GM crosstalk.

The study exclusively employs C57BL/6J mice, neglecting genetic diversity's impact on GM-host interactions, which may limit extrapolation to humans. Although taxa like *Lachnospiraceae_NK4A136_group* (Men et al., 2025, figure 4F) and *Prevotellaceae_NK3B31_group* were enriched post-CWS, their functional contributions to SCFA production (e.g., butyrate) or bile acid metabolism remain unmeasured. The original study missed a critical opportunity to link microbial shifts to quantifiable metabolic outcomes—such as luminal SCFA concentrations or serum bile acid profiles—which are essential to establish causal roles in obesity amelioration. Furthermore, as outlined in Figure 1, while the AMPK/SIRT1/PGC-1α pathway was linked to adipose browning, without knockout models, the AMPK pathway's role remains associative—not causative—in mediating cold-specific benefits. Notably, PRKAA1 upregulation (Men et al., 2025, Figure 2I) in CWS mice could reflect exercise-induced metabolic adaptation rather than cold-specific effects. Contrary to Men et al.'s claims, Luo et al. (2019) reported increased LPS and TNF-α after acute cold exposure—highlighting that CWS benefits may depend on acclimatization duration. This contrast underscores the need for time-course studies. To disentangle these stimuli, we suggest: (1) comparative models of cold exposure alone vs. swimming at neutral temperatures; (2) adipose-specific *PRKAA1* knockout mice undergoing CWS to test pathway necessity. Without such controls, the purported 'cold-specific' mechanism remains conflated with general exercise benefits.

## Translational hurdles

Beyond genetic homogeneity, translating CWS benefits to humans faces three hurdles: first, human thermogenic responses vary by age, adiposity, and acclimatization history, potentially blunting CWS efficacy. Second, human GM diversity exceeds murine models, complicating FMT engraftment predictability. Third, CWS as a sustained behavioral intervention poses adherence challenges—particularly given the psychological stress of cold exposure—which were unaddressed in the original study. Feasibility studies assessing dropout rates and stress biomarkers (e.g., cortisol) in human trials are essential next steps.

## FMT safety and ecological dynamics

Although FMT replicated CWS benefits, long-term risks such as dysbiosis or immune activation were unaddressed. Notably, recipient mice received FMT after antibiotic pretreatment—a protocol that alters mucosal immunity and may artificially enhance donor microbiota engraftment. Recent work emphasizes that FMT efficacy depends on donor-recipient microbial compatibility and host metabolic context (Marascio et al., 2023), yet Men et al. did not monitor post-FMT microbiota stability or host transcriptional adaptations. Longitudinal studies tracking GM dynamics and host responses are critical to assess clinical feasibility. To address safety concerns, future FMT studies should incorporate: (1) Longitudinal screening for dysbiosis (via Shannon index dynamics); (2) Immune activation markers (e.g., fecal calprotectin, serum IL-6); (3) 90-day post-FMT monitoring for metabolic rebound. Preclinical models using humanized microbiota mice could further evaluate donor-recipient compatibility prior to clinical trials.

## Data interpretation and future directions

The paradoxical Bacteroidota/Firmicutes (B/F) ratio (Men et al., 2025, figure 3J) in CWS and FMT groups underscores the limitations of phylum-level metrics. Metagenomic functional profiling (e.g., KEGG pathways) would more meaningfully link GM to host metabolism than taxonomic ratios. Additionally, HFD-induced inflammation reduction via CWS was attributed to GM changes. While suppressed hepatic TNF-α (Men et al., 2025, figure 2J) was attributed to GM changes, exercise alone reduces inflammation through myokine signaling (e.g., IL-6). The original study did not isolate cold's contribution, necessitating cytokine arrays in CWS vs. exercise-only controls. We recommend an integrated multi-omics framework: (1) Metatranscriptomics to identify bacterial genes upregulated by CWS (e.g., butyrate synthesis enzymes); (2) Metabolomics of cecal content to quantify SCFAs/bile acids; (3) Host proteomics of adipose tissue to map AMPK pathway dynamics. This triad could resolve mechanism specificity.

## Discussion

**Figure 2:**
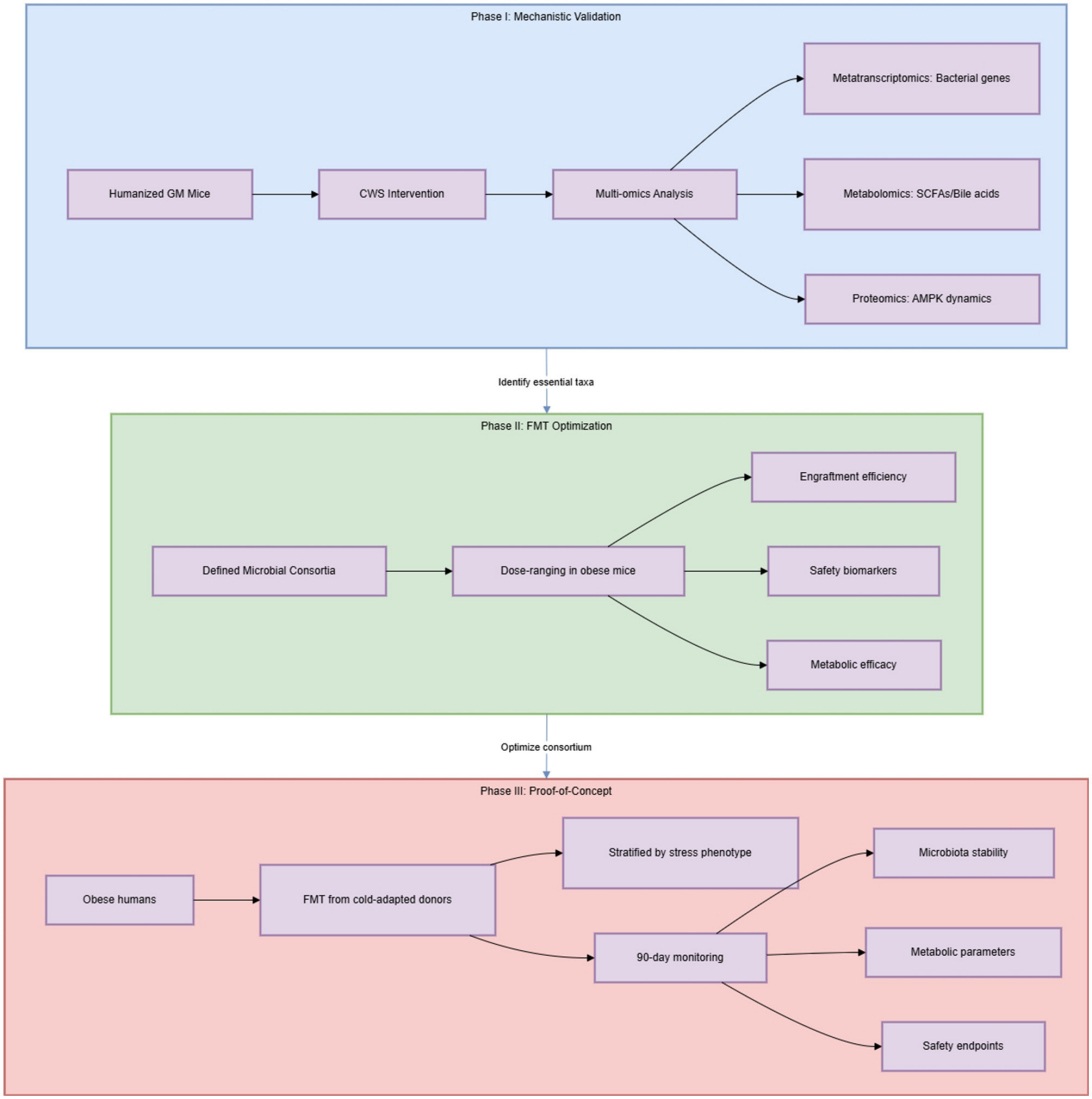
Translational research framework.

Men et al. provide compelling evidence that GM mediates CWS-induced metabolic benefits, yet key gaps remain. Prioritizing functional validation of candidate bacteria, resolving cold vs. exercise contributions to pathway activation, and evaluating FMT safety in heterogeneous populations are essential next steps. Addressing these challenges will bridge mechanistic insights to clinically actionable interventions for obesity. Addressing the psychological and physiological feasibility of CWS in humans—including stress management protocols and adherence support—is equally critical to moving beyond lab animals. A three-phase framework is proposed: (I) Mechanistic validation: Humanized GM mice + CWS to identify essential taxa; (II) FMT optimization: Dose-ranging studies with defined microbial consortia; (III) Proof-of-concept trials: Obese humans receiving FMT from cold-adapted donors, with stringent safety monitoring and stress phenotyping, as outlined in Figure 2.

## References

[B1] Luo B. Shi H. Zhang K. Wei Q. Niu J. Wang J.. (2019). Cold stress provokes lung injury in rats co-exposed to fine particulate matter. Ecotoxicol. Environ. Saf. 168, 9–16. 10.1016/j.ecoenv.2018.10.064 30384172

[B2] Marascio N. Scarlata G. G. M. Romeo F. Cicino C. Trecarichi E. M. Quirino A.. (2023). The role of gut microbiota in the clinical outcome of septic patients: state of the art and future perspectives. Int. J. Mol. Sci. 24:9307. 10.3390/ijms24119307 37298258 PMC10252956

[B3] Men J. Cui C. Li H. Li Z. Zhang Y. Liu Z.. (2025). Cold water swimming reshapes gut microbiome to improve high-fat diet-induced obesity. Front. Microbiol. 16:1589902. 10.3389/fmicb.2025.1589902 40400677 PMC12093064

